# Polymeric glabrescione B nanocapsules for passive targeting of Hedgehog-dependent tumor therapy *in vitro*

**DOI:** 10.2217/nnm-2016-0388

**Published:** 2017-03-21

**Authors:** Cinzia Ingallina, Pedro M Costa, Francesca Ghirga, Rebecca Klippstein, Julie T Wang, Simone Berardozzi, Naomi Hodgins, Paola Infante, Steven M Pollard, Bruno Botta, Khuloud T Al-Jamal

**Affiliations:** 1 Institute of Pharmaceutical Science, Faculty of Life Sciences & Medicine, King's College London, Franklin−Wilkins Building, London, SE1 9NH, UK; 2 Dipartimento di Chimica e Tecnologie del Farmaco, Sapienza Università di Roma, Rome, Italy; 3 Center for Life NanoScience@Sapienza, Istituto Italiano di Tecnologia, Rome, Italy; 4 MRC Centre for Regenerative Medicine, University of Edinburgh, Edinburgh bioQuarter, 5 Little France Drive, Edinburgh EH16 4UU, UK

**Keywords:** glabrescione B (GlaB), Hedgehog (Hh) pathway, oil-cored polymeric nanocapsules

## Abstract

**Aim:**

With the purpose of delivering high doses of glabrescione B (GlaB) to solid tumors after systemic administration, long-circulating GlaB-loaded oil-cored polymeric nanocapsules (NC-GlaB) were formulated.

**Materials & methods:**

Synthesis of GlaB and its encapsulation in nanocapsules (NCs) was performed. Empty and GlaB-loaded NCs were assessed for their physico-chemical properties, *in vitro* cytotoxicity and *in vivo* biodistribution.

**Results:**

GlaB was efficiently loaded into NCs (∽90%), which were small (∽160 nm), homogeneous and stable upon storage. Further, GlaB and NC-GlaB demonstrated specific activities against the cancer stem cells. Preliminary studies in tumor-bearing mice supported the ability of NC to accumulate in pancreatic tumors.

**Conclusion:**

This study provides early evidence that NC-GlaB has the potential to be utilized in a preclinical setting and justifies the need to perform therapeutic experiments in mice.

Cancer stem cells (CSCs) represent a subpopulation of stem cells within a tumor which drive growth and metastasis. While chemotherapy and radiation therapy are frequently able to eradicate most of the tumor bulk and proliferative cells, these treatments frequently fail in eradicating the quiescent CSCs, which might be dormant for years [1]. Eradication of the CSCs is thought to be essential to achieve complete tumor ablation and prevent cancer relapse [2]. Several signaling pathways that regulate normal stem cell self-renewal cause neoplastic proliferation when dysregulated by mutations [3]. Among those is the Hedgehog (Hh) signaling pathway, which is essential for tissue development and stemness, and whose dysregulation leads to tumorigenesis [4]. Aberrant activation of the Hh pathway is responsible for the tumorigenesis of several human cancers including medulloblastoma (MB) [5], rhabdomyosarcoma [6,7], melanoma [8], basal cell carcinoma (BCC) [9], breast [10], liver [11], lung [12,13], prostate [14,15], brain and pancreas tumors [16–19]. Thus, this pathway has been widely targeted in recent years for the development of anticancer drugs. A recent study from the co-authors of this paper identified glabrescione B (GlaB) [20], a naturally occurring isoflavone in *Derris glabrescens* [21], as the first small molecule that binds the Hh modulator Gli1 (a zinc finger) and impairs its activity by interfering with Gli–DNA interaction. GlaB was shown to inhibit the *in vitro* and *in vivo* growths of multiple Hh-dependent cancer cells and CSCs, as well as the self-renewal ability and clonogenicity of tumor-derived stem cells [22]. In this previously reported study, the ability of GlaB against MB in the allograft model of MB cells by subcutaneous injection, using a solution of GlaB (75 μmol/kg) prepared in 2-hydroxypropyl-β-cyclodextrin:ethanol (3:1), was assessed. During the 18-day treatment, GlaB significantly suppressed the tumor mass compared with the control. Despite the promising findings confirming the therapeutic efficacy of GlaB against Hh-dependent cancer, it was concluded that delivery approaches are required to solubilize GlaB in aqueous solvents so that systemic administration of relatively high doses is feasible.

Nanotechnology-based drug delivery systems offer the possibility of solubilizing poorly soluble drugs, protecting labile molecules and improving on tissue biodistribution [23]. Among the different drug delivery technologies, polymeric nanocapsules (NCs) have attracted growing attention due to the high loading capacity of hydrophobic molecules, as well as long-term stability, offering enhanced bioavailability of the active compound [24,25]. NCs consist of a core-shell structure made of hydrophilic polymer stabilized with lipophilic and/or hydrophilic surfactants surrounding an oily core, in which active molecules are confined [26]. Their preparation is generally cheap, fast and easily scalable.

This work focused on developing GlaB-loaded oil-cored polymeric NCs to improve its water solubility, so that it targets Hh-dependent cancer cells *in vitro* and *in vivo*. The choice of this formulation was encouraged by our previous studies for the delivery of water-insoluble drugs such as quercetin [27] and curcumin [28]. Physico-chemical properties, including hydrodynamic diameter, polydispersity index (PDI), ζ potential and stability, were determined. Once the desired formulation was identified, the anticancer activity of both GlaB and NC-GlaB was evaluated *in vitro* against a range of CSCs and non-CSCs, which have been reported to have a strong dependence on the Hh pathway. Finally, the *in vivo* biodistribution profile and organ histology of intravenously administered NC-GlaB was assessed in pancreatic-tumor-bearing mice. Overall, this study provides early evidence that NC-GlaB has the potential to be utilized in a preclinical setting, aiming at its future use in clinic.

## Materials & methods

### Materials

SnakeSkin™ (10K molecular weight cut-off [MWCO] dialysis tubing) was acquired from ThermoFisher (CA, USA); soybean lecithin (Epikuron 140 V) was a kind donation from Cargill Pharmaceuticals (MN, USA). Castor oil, Tween® 80, methylene chloride, absolute ethanol, DMSO and acetone were acquired from Sigma-Aldrich (UK). Bovine serum albumin solution 7.5% and 50 mM β-mercaptoethanol were purchased from ThermoFisher Scientific (UK). 75/25 DL-lactide/glycolide conjugate (PLGA–COOH, M_w_ ≈ 18,000) was a gift from Purac Biomaterials (The Netherlands). PLGA–NH–PEG and PLGA–NH–PEG–NH–DTPA were synthesized as we previously described [27,28]. The radioactivity tracer [^111^In]Cl_3_ (aqueous solution) was obtained from Convidien UK Commercial LTD (UK) and was used without prior purification. TLC strips for radiolabeling were acquired from Agilent Technologies UK Ltd (UK) and flow cytometry tubes were obtained from VWR (UK).

### Synthesis of GlaB

#### 2′-hydroxy-4′,6′-dimethoxyacetophenone **1**

A flame-dried flask was charged with 2′,4′,6′-trimethoxyacetophenone (5 g, 23.8 mmol; Sigma-Aldrich 630594) and dry CH_2_Cl_2_ (75 ml) under argon. BBr_3_ (15 ml, 87.3 mmol) was added drop wise. The resulting solution was stirred at room temperature for 2 h before adding 4 M NaOH (90 ml) and allowed to stand for 30 min. The solution was extracted with CH_2_Cl_2_ and combined organic layers were washed with brine, dried over Na_2_SO_4_ and finally concentrated under reduced pressure. The resulting solid was recrystallized from EtOH and white crystals of 2′-hydroxy-4′,6′-dimethoxyacetophenone **1** (4.43 g) were obtained.

White solid (yield 98%); melting point (mp) 81–82°C; ^1^H NMR (400 MHz, acetone-d*_6_*, δ): 13.8 (s, 1H, OH), 5.94 (d, J = 2.4 Hz, 1H, Ar H), 5.91 (d, J = 2.4 Hz, 1H, Ar H), 3.80 (s, 3H, OCH_3_), 3.72 (s, 3H, OCH_3_), 2.43 (s, 3H, CH_3_); ^13^C NMR (100 MHz, acetone-d*_6_*, δ): 202.5 (C=O), 167.36 (C4), 166.34 (C6), 163.15 (C2), 105.5 (C1), 93.42 (C3), 90.43 (C5), 55.20 (OCH_3_), 55.00 (OCH_3_), 31.94 (CH_3_); IR (KBr): ν = 3100, 1614, 1271 cm^-1^; high-resolution mass spectrometry (HRMS; ESI) m/z: [M+H]^+^ calculated for C_10_H_12_O_4_+H^+^, 197.080800; found, 197.080848.

#### 3-dimethylamino-1-(2-hydroxy-4,6-dimethoxyphenyl) propenone **2**

A mixture of 2′-hydroxy-4′,6′-dimethoxyacetophenone **1** (4.5 g, 22.9 mmol) and N,N-dimethylformamide dimethylacetal (13 ml, 97.1 mmol) was heated at 95°C for 3 h. Afterward, the solvent was removed under reduced pressure and the obtained **2** (5.8 g) was used in the subsequent step without any further purification.

Red solid (quantitative yield); mp 145–147°C; ^1^H NMR (400 MHz, CDCl_3_, δ): 15.65 (s, 1H, OH), 7.92 (d, J = 12 Hz, 1H, =CH–N), 6.25 (d, J = 12.0 Hz, 1H, =CH–(CO), 6.07 (d, J = 2.4 Hz, 1H, Ar H), 5.91 (d, J = 2.4 Hz, 1H, Ar H), 3.84 (s, 3H, OCH_3_), 3.80 (s, 3H, OCH_3_), 3.15 (s, 3H, NCH_3_), 2.92 (s, 3H, NCH_3_); ^13^C NMR (100 MHz, CDCl_3_, δ): 191.64 (C=O), 169.00 (C4), 165.54 (C6), 162.86 (C2), 155.83 (CH–N), 106.58 (C1), 96.27 (CH–(CO)), 95.59 (C3), 92.06 (C5), 57.09 (OCH_3_), 56.57 (OCH_3_); IR (KBr): ν = 3447, 1614, 1542, 1362 cm^-1^; ESI–MS m/z: [M+H]^+^ calculated for C_13_H_17_O_4_N+H^+^, 252.1; found, 252.3.

#### 3-iodo-5,7-dimethoxy-4H-chromen-4-one **3**

The enamino ketone **2** was dissolved in CH_3_OH (450 ml) and I_2_ (11.7 g, 46.2 mmol) was added to the solution. The mixture was stirred at room temperature for 1 h, then the solvent was evaporated. To remove residual I_2_, the crude was treated with a saturated aqueous Na_2_S_2_O_3_ solution until the mixture became clear. The mixture was then extracted with CHCl_3_, and the combined organic layers were dried over Na_2_SO_4_ and concentrated under reduced pressure. The residue was purified by column chromatography using hexane–EtOAc as eluent to obtain 3-iodo-5,7-dimethoxy-4H-chromen-4-one **3** (2.5 g) as a white powder.

White solid (yield 33%); mp 156–157°C; ^1^H NMR (400 MHz, CDCl_3_, δ): 8.02 (s, 1H, H-2), 6.37 (d, J = 2.0 Hz, 1H, Ar H), 6.32 (d, J = 2.0 Hz, 1H, Ar H), 3.87 (s, 3H, OCH_3_), 3.82 (s, 3H, OCH_3_); ^13^C NMR (100 MHz, CDCl_3_, δ): 183.43 (C=O), 164.28 (C7), 160.97 (C5), 159.81 (C9), 155.33 (C2), 107.53 (C10), 96.59 (C8), 92.44 (C6), 89.71 (=C–I), 56.41 (OCH_3_), 55.79 (OCH_3_); IR (KBr): ν = 1671, 1640, 1600–1450 cm^-1^; HRMS (ESI) m/z: [M+H]^+^ calculated for C_11_H_9_O_4_I+H^+^, 332.961800; found, 332.961633.

#### 3-(3′,4′-methylendioxyphenyl)-5,7-dimethoxy-4H-chromen-4-one **4**

To a solution of **3** (2.5 g, 7.5 mmol), in 1,2-dimethoxyethane/H_2_O = 50:50 (150 ml) were added Na_2_CO_3_ (3.18 g, 30 mmol), 3,4-(methylenedioxy)-phenylboronic acid (1.8 g, 11 mmol) and Pd EnCat™40 (937 mg, 5%). The resulting mixture was stirred at 45°C for 2 h and then filtered. The catalyst was washed with H_2_O and CH_2_Cl_2_. The aqueous phase was extracted with CH_2_Cl_2_. The combined organic layers were dried over Na_2_SO_4_ and concentrated under reduced pressure. The crude residue was purified by flash chromatography using hexane–EtOAc as eluent to give **4** (1.4 g) as a gray powder.

Gray solid (yield 57%); mp: 155–156°C; ^1^H NMR (400 MHz, CDCl_3_, δ): 7.75 (s, 1H, H-2), 7.1 (d, J = 2.0 Hz, 1H, H-2′), 6.94 (dd, J = 8.0 and 2.0 Hz, 1H, H-6′), 6.83 (d, J = 8.0 Hz, 1H, H-5′), 6.44 (d, J = 2.2 Hz, 1H, H-6), 6.37 (d, J = 2.2 Hz, 1H, H-8), 5.97 (brs, 2H, O–CH_2_–O), 3.94 (s, 3H, OCH_3_), 3.89 (s, 3H, OCH_3_); ^13^C NMR (100 MHz, CDCl_3_, δ): 175.09 (C=O), 164.02 (C7), 161.54 (C5), 160.30 (C9), 150.05 (C2), 147.58 (C3′), 147.58 (C4′), 126.5 (C3), 126.04 (C1′), 122.84 (C6′), 110.50 (C2′), 110.0 (C10), 108.35 (C5′), 101.30 (O–CH_2_–O), 96.74 (C6), 92.73 (C8), 56.41 (OCH_3_), 55.79 (OCH_3_); IR (KBr): ν = 1654, 1456, 1247 cm^-1^; HRMS (ESI) m/z: [M+H]^+^ calculated for C_18_H_14_O_6_+H^+^, 327.086300; found, 327.086206.

#### 3-(3′,4′-dihydroxyphenyl)-5,7-dimethoxy-4H-chromen-4-one **5**

A mixture of **4** (1.4 g, 4.3 mmol) and Pb(OAc)_4_ (7.5 g, 17 mmol, freshly recrystallized from AcOH) in dry C_6_H_6_ (100 ml) was stirred at 80°C under argon overnight. After, being cooled to room temperature, the reaction mixture was filtered through a pad of Celite, washed with CH_2_Cl_2_ and concentrated under reduced pressure. The crude was diluted with THF/H_2_O = 5:1 (50 ml) and CH_3_COOH (50 ml), and the resulting mixture was stirred at room temperature for 6 h. Afterward, a saturated aqueous NaHCO_3_ solution was added until pH 8 and extracted with EtOAc. To the combined organic layers was added a solution of NaOH 0.1 M. Water layer was treated with CH_3_COOH and then extracted with EtOAc. The combined organic layers were dried over Na_2_SO_4_ and concentrated under reduced pressure to obtain **5** (570 mg).

Yellow solid (yield 42%); mp: 127–129°C; ^1^H NMR (400 MHz, CH_3_OD): 7.86 (s, 1H, H-2), 6.88 (brs, 1H, H-2′), 6.70 (brs, 2H, H-5′ and H-6′), 6.50 (d, J = 2.0 Hz, 1H, H-6), 6.40 (d, J = 2.0 Hz, 1H, H-8), 3.80 (s, 3H, OCH_3_), 3.79 (s, 3H, OCH_3_); ^13^C NMR (100 MHz, CH_3_OD, δ): 175.00 (C=O), 165.08 (C7), 161.13 (C5), 159.91 (C9), 151.28 (C2), 145.12 (C3′), 145.0 (C4′), 126.49 (C3), 123.12 (C1′), 120.50 (C6′), 116.99 (C2′), 110.00 (C10), 114.48 (C5′), 95.86 (C6), 92.82 (C8), 55.09 (2 × OCH_3_); IR (KBr): ν = 3376, 1637 cm^-1^; HRMS (ESI) m/z: [M+H]^+^ calculated for C_17_H_14_O_6_+H^+^, 315.086300; found, 315.086363.

3-(3′,4′-bis(3-methylbut-2-enyloxy)phenyl)-5,7-dimethoxy-4H-chromen-4-one; GlaB. To a solution of **5** (570 mg, 61.8 mmol) in acetone (100 ml) was added K_2_CO_3_ (7.4 g, 5.4 mmol) and, after 10 min of stirring at room temperature, was added 3,3-dimethylallyl bromide (968 mg, 6.5 mmol). Then, the mixture was stirred at 80°C overnight. Afterward, the solvent was evaporated. The resulting solid was dissolved in EtOAc and extracted with water. The combined organic layers were dried over Na_2_SO_4_ and finally concentrated under reduced pressure. The crude was purified by column chromatography using hexane–EtOAc as eluent to obtain GlaB as white powder. The powder was recrystallized from hexane resulting with white crystals.

White crystal (yield 90%); mp 102–104°C. The spectral data of this product were identical to an authentic sample of GlaB.

### Formulation of the NCs

#### Encapsulation protocol

**Figure 1.**
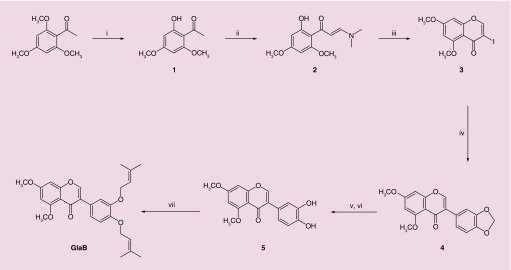
**Synthesis of Glabrescione B.** Reagents and conditions: **(i)** boron tribromide, dry dichloromethane, RT, 2 h; **(ii)** DMF–DMA, 95°C, 3 h; **(iii)** iodine, methanol, RT, 1 h; **(iv)** 3,4-(methylenedioxy)-phenylboronic acid, PdEnCat™ 40, sodium carbonate, DME/H_2_O 1:1 (v/v), 45°C, 2 h; **(v)** lead tetracetate, benzene dry, 80°C, overnight; **(vi)** acetic acid, THF/H_2_O (5:1 v/v), RT, 1 h; **(vii)** 3,3-dimethylallyl bromide, potassium carbonate, acetone, reflux, 72 h. RT: Room temperature.

NC-GlaB were prepared by using three poly(lactic-*co*-glycolic acid) (PLGA) polymer derivatives: the commercially available PLGA, PEGylated PLGA (PLGA–PEG) conjugates capable of prolonged blood circulation profile and PLGA–PEG conjugated with diethylene triamine pentaacetic acid (DTPA; PLGA–PEG–DTPA), which allows the tracking of NCs *in vivo* by γ scintigraphy. NCs containing GlaB were prepared using the nanoprecipitation method described by Fessi *et al*. (Figure 1) [29]. Briefly, 2.5 ml of acetone/ethanol (60:40 v/v) containing polymer (the commercially available PLGA [100%], PLGA–NH–PEG [100%] or PLGA–NH–PEG/PLGA–NH–PEG–DTPA [90%:10%]) (12.5 mg), castor oil (75 μl), GlaB (4 mg) and soybean lecithin (12.5 mg) was added dropwise into 5 ml of aqueous phase (containing 0.2% of hydrophilic surfactant Tween^®^ 80). The mixture was stirred in the hood for 30 min to allow solvent diffusion and NC formation. Subsequently, the organic solvents were removed via rotatory evaporation under reduced pressure in a Buchi Rotavapor^®^ (Buchi, UK). Finally, the volume of suspension was adjusted to 5 ml.

#### Solubility of GlaB in castor oil

The solubility of GlaB in castor oil was evaluated using the simple saturation shake-flask method [30]. Briefly, excess GlaB (10 mg) was transferred to vials containing 1 ml of castor oil, sealed and incubated at 37°C for 48 h with shaking (250 strokes/min). Subsequently, 100 μl of the supernatant was diluted in ethanol, and the amount of GlaB dissolved in the organic solvent was determined using a UV–Vis spectrophotometer (UV-1601 PC, Shimadzu, Japan) by measuring the absorbance at 258 nm, against a GlaB standard curve.

#### Determination of GlaB encapsulation efficiency

To purify the sample from un-encapsulated drug, NCs were passed through a PD10 desalting column (GE Healthcare). To disrupt the NCs and quantify the total amount of drug in NCs prior and after purification, NC preparations (30 μl) were diluted in ethanol (to a final volume of 2 ml). Subsequently, the amount of GlaB in NCs was determined using a UV–Vis spectrophotometer (UV-1601 PC, Shimadzu) by measuring the absorbance at 258 nm against a standard plot of known GlaB concentrations. The amount of GlaB in the measured samples was then calculated from the standard plots (Supplementary materials) and the encapsulation efficiency (EE, %) was calculated using the following equation:
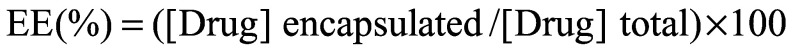



Drug loading efficiency (% LE) was calculated using the following equation:
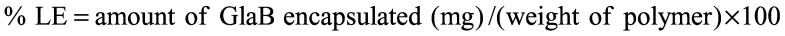



### Physico-chemical characterization of NCs

#### Size & ζ potential

The average size and ζ potential of the NCs were measured by dynamic light scattering with a Nanosizer ZS Series (Malvern Instruments, MA, USA), using disposable polystyrene cells and plain-folded capillary Zeta cells. Dilutions were prepared in deionized water and measurements performed at room temperature. The electrophoretic mobility was used to calculate the ζ potential using the Helmholtz–Smoluchowski equation. Results for the hydrodynamic size are presented as the average of 20 measurements, each performed in triplicate.

#### Release profile & serum stability in vitro

About 2 ml of NC formulation containing 2 mg (GlaB) in 5 ml was transferred into a 10 kDa dialysis bag, and dialysis was performed against 200 ml of phosphate-buffered saline (PBS; pH 7.4) containing 10% (w/v) Tween^®^ 80, at 37°C under stirring (250 strokes/min). Samples were taken after 24 h, and drug concentration was determined using a Perkin-Elmer Lambda 35 UV–Vis spectrophotometer by measuring the absorbance at 258 nm.

#### Shelf life stability

NC suspensions were transferred to 7 ml glass vials, sealed and stored at 4°C. The stability of NCs was tested after 0, 7, 14 and 28 days by visual inspection (color and opacity) and by measuring the hydrodynamic size and ζ potential. Results of measurements (n = 3) are presented as average ± standard deviation (SD).

#### Cell culture

SKOV3 human ovary adenocarcinoma cell line was cultured in McCoy media, while PANC1 human pancreatic carcinoma cell line and GL261 murine glioma cell line were cultured in Advanced Roswell Park Memorial Institute (RPMI) media (Sigma, MO, USA), at 37°C in 5% CO_2_. Culture medium was supplemented with 10% fetal bovine serum (FBS), 100 U/ml penicillin, 100 μg/ml streptomycin and 1% l-glutamine (Sigma). Cells were routinely grown in 75 cm^2^ tissue culture flasks and passaged twice a week, after detachment with trypsin/EDTA (Sigma), when reaching 80% confluency.

Human (G7) and mouse glioma-initiating neural stem cell line (IENS) glioblastoma (GBM), as well as their culturing conditions, have been described previously [31–34]. Briefly, cells were cultured using serum-free complete media (described in Supplementary materials) supplemented with B27 (Life Technologies, UK, 17504044) and N2 (Life Technologies, 17502048). Growth factors EGF (Peprotech, UK, 315–09–500) and FGF-2 (Peprotech, 100–18B-500) (10 ng/ml), as well as laminin (Sigma, L2020, 1 μg/ml), were added freshly before cell split. Cells were routinely grown to confluence and split typically twice per week after dissociation with Accutase solution (Sigma, A6964) and centrifugation. Differentiation of G7 cells was performed as described previously by Pollard and collaborators [35]. After reaching 70% confluency, G7 cells were washed with PBS and replaced with media containing bone morphogenetic protein (BMP)4 (Peprotech 120–05ET-10, 10 ng/ml), but no EGF or FGF-2. Differentiation media, containing BMP, was replaced every 7 days throughout the time course and supplemented with fresh BMP at 4 days.

#### Cytotoxicity studies in vitro

Cells seeded in 96-well plates were incubated with GlaB or NC-GlaB (200 μl final volume in complete media) for 48, 72 and 96 h. Subsequently, the media was removed and replaced with 120 μl of MTT solution (0.5 mg/ml). Cells were incubated for 3 h in normal culture conditions, after which the formazan crystals were dissolved in DMSO (200 μl) and sample absorbance at 570 nm was measured in a FLUO star OPTIMA plate reader (BMG Labtech, UK). The results, expressed as the percentage cell survival (mean ± SD), were calculated using the following equation: % cell survival = (A570 nm of treated cells/A570 nm of untreated control cells) × 100.

#### Cell cycle analysis by flow cytometry

The percentage of cells in each phase of the cell cycle was determined by flow cytometry. Briefly, cells seeded in 24-well plates (5 × 10^4^) were treated with 5 μM of GlaB, NC-GlaB or equivalent concentrations of empty NCs. After treatment, cells were washed with PBS, trypsinized and transferred into BD flow cytometer tubes. After centrifugation at 500 × *g* for 5 min (for trypsin removal), cells were fixed by incubation with 70% cold ethanol for 1 h. After washing with PBS (to remove traces of ethanol), fixed cells were treated with 50 μl of RNase A in PBS (100 μg/ml, 10 min at 37°C), to ensure that RNA labeling does not interfere with the results. Subsequently, cells were stained for 30 min at 37°C in the dark with 400 μl of propidium iodide (PI) solution (40 μg/ml of PI in PBS). PI fluorescence was then measured in the FL-2 channel using a BD FACS Calibur flow cytometer (BD Bioscience, CA, USA). For each condition, 10,000 events were gated and fluorescence was analyzed using the FlowJo software. Results were expressed as averages of percentage cell populations in each phase of the cell cycle ± SD (n = 3).

#### Western blot analysis

SKOV3, PANC1, GL261, G7 and IENS GMB cells were cultured on 6-well plates and incubated with culture media for 24 h. PANC1 and SKOV3 cells were cultured on 6-well plates and incubated with 5 μM GlaB for 24 or 48 h. At the end of the treatment, cells were rinsed twice with ice-cold PBS, incubated with RIPA buffer (radioimmunoprecipitation Assay buffer, 1% triton X-100, 0.5% sodium deoxycholate, 0.1% SDS, 50 mM Tris-HCl and 0.15 M NaCl, pH 8) containing protease inhibitor (Complete Ultra, Roche Diagnostics GmbH, Germany). Cells were scrapped out and the collected lysates were kept on ice for additional 30 min with intermittent vortexing every 10 min. Lysate solutions were centrifuged at 17,949 × *g* for 30 min, and the supernatants containing cell proteins were collected. The concentration of the isolated proteins was determined using BCA Protein Assay kits (Pierce BCA protein assay kit, Thermal Scientific, UK). About 100–200 μg of the protein was resolved in 8% SDS-PAGE and electrophoretically transferred to Hybond ECL nitrocellulose membranes (GE Healthcare, UK). Membranes were blocked in 3% bovine serum albumin (Sigma-Aldrich, UK) at room temperature for 1 h. Membranes were then incubated with the primary antibodies against Gli1 (rabbit antimouse antibody, PA5–23411; rabbit antihuman antibody, PA5–28384; Thermofisher Scientific, UK) at 1:1000 dilution or GAPDH (#14C10, Cell Signaling Technology, CA, USA) at 1:5000 dilution over night at 4°C. Membranes were washed three-times with Tris-buffered saline containing 0.1% Tween^®^ 20 (TBS-T) and then incubated with secondary antibodies, horseradish peroxidase-linked goat antirabbit antibodies (#7074, Cell Signaling Technology) at 1:3000 dilution for 2 h at room temperature. The protein bands were detected using chemiluminescent kits (Immun-Star™ Chemiluminescent Kit, BioRad, UK) and imaged by the ChemiDoc MP imaging system and Image Lab software (BioRad). GAPDH was used as an internal protein control. Values for Gli1 were normalized to the total GAPDH and then to untreated IENS, in the case of treatment to untreated PANC1 and SKOV3.

### Radiolabeling of NCs

#### Radiolabeling of NC–PEG–DTPA–GlaB

The radioactive probe ^111^InCl_3_ was purchased from Mallinckrodt Pharmaceuticals (The Netherlands) as an aqueous solution in 0.5 M HCl and used without further purification. To radiolabel the NC with indium-111, NCs were prepared in water as described in the previous section except that PLGA–PEG–DTPA was included at 10% (w/w) of total polymer content and concentrated 28-times using rotavap, so that the final drug and polymer concentrations are 1.22 and 71.43 mg/ml, respectively. The NC suspension (700 μl) was incubated with 2 M ammonium acetate (one-ninth of the reaction volume, pH 5.5), to which 10 MBq of ^111^InCl_3_ was added per injection dose for γ scintigraphy. The reaction was kept at room temperature for 30 min with intermittent vortexing every 10 min. Upon completion, the radiolabeling reaction was quenched by the addition of 0.1 M EDTA chelating solution (one-twentieth of the reaction volume). ^111^InCl_3_ alone and NCs without DTPA incorporation were also subjected to the same conditions of the labeling reaction as a control.

#### Radiolabeling efficiency & serum stability studies

To evaluate the radiolabeling efficiency, the labeled NC-^111^In was spotted on the TLC strips, which were developed in 0.1 M ammonium acetate containing 25 mM EDTA as a mobile phase. Strips were allowed to dry before being developed and counted quantitatively using a cyclone phosphor detector (Packard Biosciences, UK). The NC-^111^In was passed through PD-10 column before injecting into animals to exchange the ammonium acetate buffer (pH 5.5) with PBS (pH 7.4) and to ensure that no free ^111^In-EDTA was present in the injected dose. The sample was collected from the column and the radiolabeling efficiency was examined immediately after the collection. As a result, the sample was further diluted two-times to three-times compared with the original sample. Final drug concentration after filtration was 1.22 mg/ml. To determine the stability of the radiolabeled NC-^111^In, 20 μl of the sample was incubated with 20 μl of serum and 20 μl of PBS at 37°C. At 24 h, 2 μl of the incubated samples was spotted to the TLC strips, which were developed and quantified as described above. Free ^111^In-EDTA was detected at the solvent front while radiolabeled NC was retained at the application point. Indium-labeled NC without DTPA incorporation was included as a control.

#### Tumor xenograft animal model studies

All animal experiments were performed in compliance with the UK Home Office Code of Practice for the Housing and Care of Animals Used in Scientific Procedures. Six- to eight-week-old female SCID/Beige mice (Harlan Laboratories, UK) were caged in individually vented cages in groups of four with free access to food and water. A temperature of 19–22°C was maintained, with a relative humidity of 45–65%, and a 12 h light/dark cycle. Established human pancreatic PANC1 and PANC0403 xenograft tumor models were applied. Mice were inoculated subcutaneously with 1 × 10^6^ cells in 100 μl on either flanks subcutaneously.

#### Tissue biodistribution by γ scintigraphy

Forty days after tumor inoculation, pancreatic-tumor-bearing SCID/Beige mice (n = 2 per time point) were injected intravenously, via tail vein, with 200 μl of NCs at a dose of 115 mg/kg of polymer and 18.5 mg/kg of drug containing approximately 1 MBq of radioactivity (∼2.3 mg of polymer, ∼370 μg of drug, 1 MBq per mouse). Animals were sacrificed after 30 min or 24 h. The tumors and major organs (brain, lung, liver, spleen, kidney and heart) were collected, weighed, and the ^111^In activity in the collected tissues was quantified using an automated γ counter (LKB Wallac 1282 Compugamma, Perkin Elmer, UK). A portion of the radiolabeled NCs was also analyzed as a reference. The γ counter corrected for physical radioisotope decay. Radioactivity readings (c.p.m.) were plotted as a % ID/organ or as a % ID/g of tissue.

#### Organ toxicity evaluation

Twenty-eight days after tumor inoculation, pancreatic-tumor-bearing SCID/Beige mice (n = 6) were injected, via *tail* vein, with 200 μl of the NCs at a dose of 78 mg/kg of polymer and 12.5 mg/kg of drug (∼1.56 mg of polymer and ∼250 μg of drug per mouse) once a week for 3 weeks. At the experiment end point, the mice were sacrificed and the major organs (liver, lung, tumor, heart, kidney and spleen) were fixed in 10% neutral buffer formalin as 5 mm^2^ pieces. The organs were then paraffin-embedded and sectioned for hematoxylin and eosin according to the standard histological protocols at the Royal Veterinary College. The stained sections were analyzed with a Leica DM 1000 LED Microscope (Leica Microsystems, UK) coupled with charge-coupled device (CDD) digital camera (Qimaging, UK).

## Results

### Synthesis of GlaB

The synthesis of the GlaB (Figure 1) began from commercially available 2′,4′,6′-trimethoxyacetophenone. The O-demethylation was selectively obtained in position 2 by treatment of 2′,4′,6′-trimethoxyacetophenone with boron tribromide (BBr_3_) to give 2′-hydroxy-4′,6′-dimethoxyacetophenone **1**. Compound **1** was subsequently treated with N,N-dimethylformamide dimethyl acetal to form the enamino ketone **2**, which, on stirring in methanol with excess iodine, underwent tandem cyclization and iodination to afford 3-iodo-5,7-dimethoxy-4H-chromen-4-one **3** [36]. Cross-coupling of **3** with 3,4-(methylenedioxy)-phenylboronic acid in the presence of Pd EnCat™40 catalyzed Suzuki reaction gave 3-(3′,4′-methylendioxyphenyl)-5,7-dimethoxy-4H-chromen-4-one **4.** Notably, the Suzuki–Miyaura reaction was accomplished by the use of polyurea-encapsulated palladium (Pd EnCat™ 40), a robust and recyclable catalyst which was designed in 2002 [37] and used under mild conditions [38]. The methylene acetal in **4** was oxidatively removed by treatment with Pb(OAc)_4_ allowing the clean formation of acetoxy acetal, which was smoothly hydrolyzed to provide the desired catechol **5** [39]. The final step was the prenylation of compound **5**, which was accomplished by treatment with 3,3-dimethylallyl bromide and K_2_CO_3_ under refluxing acetone to yield GlaB [40]. The spectral data of GlaB obtained by synthesis were identical to those found in the natural product. The overall reaction yield was 7%.

### Formulation & physico-chemical characterization of NCs

**Figure 2.**
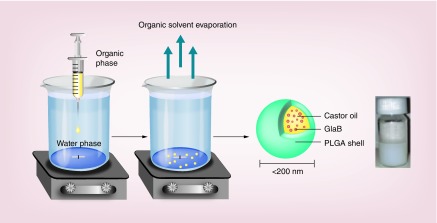
**Schematic representation of the formulation method of the oil-cored nanocapsules.** The organic phase, composed of polymer, oil, lipophilic surfactant and the drug dissolved in a water-miscible solvent, was added dropwise to an aqueous phase containing a hydrophilic surfactant. Formation of NCs takes place by solvent diffusion and polymer precipitation. Finally, the solvent was removed forming an aqueous dispersion of the NC. The average diameter of the NCs is approximately 160 nm. NC: Nanocapsule.

The preparation of non-PEG-containing NC (NC-GlaB) is described in detail in the ‘Materials & methods’ section. PEG-containing NCs (NC-PEG) were also prepared, due to their prolonged blood circulation time facilitated by PEG. All formulations were prepared using the nanoprecipitation method (Figure 2). The purified formulations were then characterized for their hydrodynamic diameter, PDI and ζ potential, immediately after purification and over 28 days storage at 4°C (Table 1 & Supplementary Table 1). Castor oil was selected as the core material due to its good oil solubilization ability of the drug (27 μg/μl at 37°C). Importantly, efficient drug encapsulation (∼90%) was achieved for both NC-GlaB and NC-PEG-GlaB (Table 1 & Supplementary Table 1). In contrast to the extremely poor aqueous solubility of GlaB [21], NC-GlaB were well dispersed and formed a homogeneous suspension in aqueous media with PDI values below 0.2 and an average hydrodynamic diameter of approximately 160 nm. All NC formulations were found to be stable over the 28-day storage period, with no significant changes in size, PDI or ζ potential observed over time. No apparent change in color or phase separation was observed in any of the formulations.

**Table 1.** **Shelf-life stability of oil-cored nanocapsules prepared using PLGA as polymer.**

**Sample**	**Day**	**Hydrodynamic diameter^†,‡^**	**PDI^†,‡^**	**ζ potential (mV)^‡,§^**	**Encapsulation efficiency (% EE)^‡,¶^**	**Loading efficiency (% LE)^‡,#^**
NC	1	164.0 ± 1.9	0.19 ± 0.01	-54.0 ± 1.8	–	–

	7	162.4 ± 1.2	0.18 ± 0.01	-59.3 ± 0.2		

	14	151.1 ± 0.8	0.14 ± 0.01	-67.8 ± 1.6		

	21	155.6 ± 1.5	0.17 ± 0.02	-56.2 ± 0.6		

	28	166.1 ± 1.3	0.21 ± 0.01	-57.6 ± 0.7		

NC-GlaB	1	159.4 ± 1.0	0.17 ± 0.01	-39.8 ± 0.5	85 ± 6.1	27.5 ± 1.9

	7	155.9 ± 0.4	0.12 ± 0.02	-47.0 ± 0.6		

	14	148.6 ± 1.0	0.13 ± 0.02	-58.0 ± 1.8		

	21	154.8 ± 0.9	0.14 ± 0.02	-49.7 ± 1.5		

	28	154.3 ± 1.1	0.14 ± 0.01	-46.7 ± 1.7		

^†^Measured with dynamic light scattering.

^‡^Expressed as mean ± SD (n = 3).

^§^Analyzed with electrophoretic light scattering using 10 mM NaCl.

^¶^Calculated as percentage of initial drug added, which was determined by spectrophotometry.

^#^Calculated as mass of incorporated drug divided by the weight of polymer, which was determined by spectrophotometry.

GlaB: Glabrescione B; NC: Nanocapsule; NC-GlaB: GlaB-loaded oil-cored polymeric nanocapsule; PDI: Polydispersity index; SD: Standard deviation.

### *In vitro* stability of the NCs

**Figure 3.**
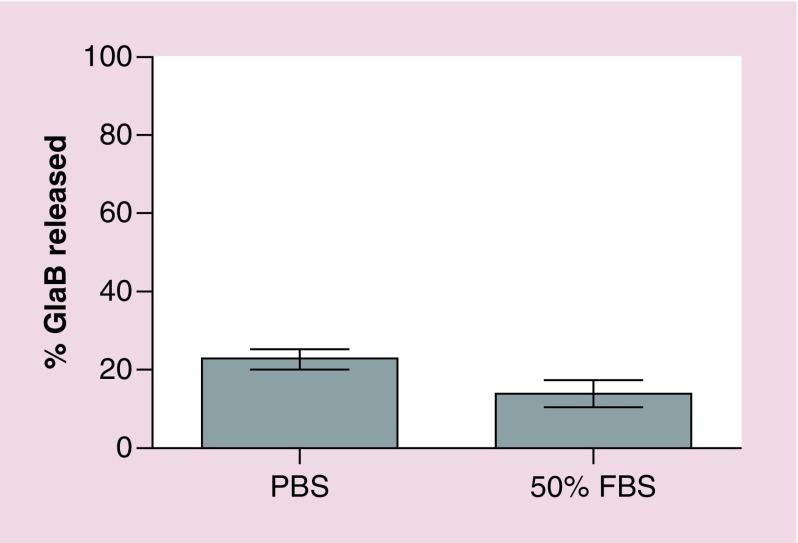
**Release profile of glabrescione B from nanocapsules.** NC–PEG–GlaB were dialyzed for 24 h against 10% w/v Tween^®^ 80 in PBS, pH 7.4, in the presence or absence of 50% FBS. Drug concentration inside the dialysis bag was assessed by measuring the absorbance at 258 nm. Results are expressed as means ± standard deviation (n = 3). FBS: Fetal bovine serum; NC-PEG-GlaB: PEG-containing nanocapsules loaded with GlaB; GlaB: Glabrescione B; PBS: Phosphate-buffered saline.

To determine the *in vitro* stability of GlaB in the NC formulation, drug release from NC–PEG–GlaB was assessed over 24 h by the dialysis method, in the presence or absence of FBS. The dialysate used was PBS pH 7.4 containing 10% w/v Tween^®^ 80 to maintain GlaB solubility in aqueous media. As shown in Figure 3, drug release from the NC formulation did not exceed 20% over the course of 24 h, a value that was not altered by the presence of 50% FBS.

### *In vitro* cytotoxicity of GlaB

The MTT assay was performed in order to estimate the toxicity of free or NC-formulated GlaB. Two different types of cell lines were selected for this study: non-stem cancer cells that contain a small CSC subpopulation (PANC1, human pancreatic carcinoma; SKOV3, human ovary adenocarcinoma; and GL261, murine glioma) [41–43] and pure glioblastoma-derived neural stem cells (GNS; patient-derived G7 human and engineered Ink4a/ARF, EGFRvIII overexpressed IENS murine) [31]. It is hypothesized that the GNS cell lines will be more sensitive to GlaB as they consist entirely of CSCs and therefore have a stronger dependence on the Hh pathway. Indeed, western blot quantification of Gli1 protein expression, a downstream effector in the Hh pathway, showed higher Gli1 protein levels in pure CSCs (IENS and G7), compared with non-stem cancer cells (PANC1, SKOV3 and GL261) (Supplementary Figure 1).

**Figure 4.**
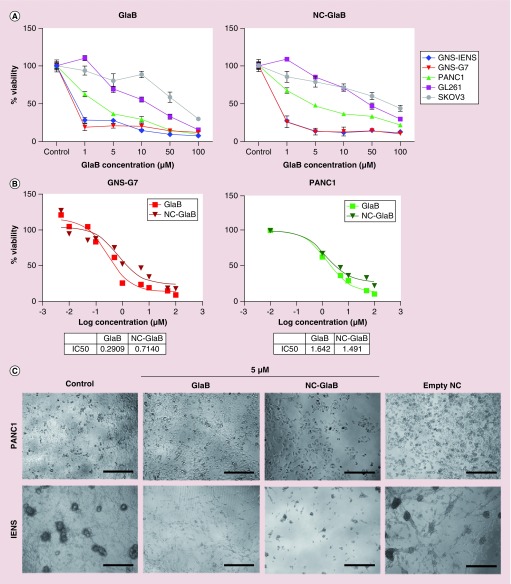
***In vitro* cytotoxicity of glabrescione B and glabrescione B-loaded oil-cored polymeric nanocapsules.** Cells were incubated with GlaB or NC-GlaB for 96 h at increasing drug concentrations (1–100 μM). **(A)** Cell viability, expressed as a percentage of control untreated cells, was determined by MTT assay after 96 h incubation with free GlaB (left) or NC-GlaB (right) treatments. **(B)** GlaB and NC-GlaB dose–response curve and IC_50_ values for GNS-G7 (left) and PANC1 cells (right) after 96 h treatment with GlaB or NC-GlaB at increasing drug concentrations (from 0.05–1 to 100 μM). Results are expressed as mean ±SD (n = 5). **(C)** Microscopic observation of cells at 96 h after treatment with GlaB, NC-GlaB or empty NC, at a final concentration of 5 μM GlaB. Scale bar corresponds to 150 μm. GlaB: Glabrescione B; NC: Nanocapsule; NC-GlaB: GlaB-loaded oil-cored polymeric nanocapsule; SD: Standard deviation.

As demonstrated in Figure 4A and Supplementary Figure 2, a dose-dependent and time-dependent reduction in cell viability was observed in both treatment conditions (NC-GlaB and free GlaB). Incubation for 96 h with 5 μM of free GlaB/NC-GlaB resulted in a significant reduction in cell viability in IENS (∼27%/14% cell viability; p < 0.05) and G7 (∼20%/13%; p < 0.001/p < 0.01) cells, while a smaller decrease was seen in GL261 (∼67%/84%; p > 0.05) or PANC1 (∼36%/48%; p > 0.05) cells. Light microscopy images obtained during treatment confirmed the dose-dependent cell killing effect verified with the MTT assay. Morphological changes, namely smaller and more round, were observed during incubation with free GlaB or NC-GlaB (Figure 4C). Cells treated with empty NCs, at equivalent concentrations to those used in NC-GlaB, showed normal cell morphology except at very high concentration, in which slight but significant toxicity was detected (Supplementary Figure 3).

IC_50_, the drug amount required to achieve 50% cell kill, was estimated in G7 and PANC1 cells. The values obtained following 96 h treatment with free GlaB/NC-GlaB were 0.29 μM/0.71 μM (G7) and 1.62 μM/1.49 μM (PANC1) (Figure 4B). The toxicity of NC–PEG–GlaB was also tested, to assess whether the incorporation of PEG into the NCs has any effect on cell viability. Comparable results were obtained at 96 h with 5 μM GlaB, for NC–PEG–GlaB (∼74%) and NC–GlaB (∼86%) (Supplementary Figure 4).

### GlaB effect on cell cycle

**Figure 5.**
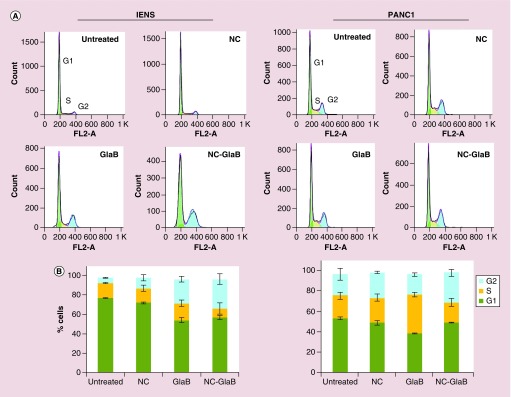
**Cell cycle analysis in cultured IENS and PANC1 cells after treatment with glabrescione B.** **(A)** Representative histograms showing the cell cycle distribution and **(B)** relative changes in the percentage of cells in each cell cycle phase, following incubation for 48 h with 5 μM GlaB. Results are expressed as mean ± standard deviation (n = 3). GlaB: Glabrescione B; NC: Nanocapsule; NC-GlaB: GlaB-loaded oil-cored polymeric nanocapsule.

Having shown that free or NC-formulated GlaB has an effect on the viability of GNS cells and, to a lesser extent, on non-stem cancer cells, experiments were performed to determine whether the effect could be due to alterations in the cell cycle mechanism. As shown in Figure 5 (left panel) and Supplementary Figure 5, a steady increase in cell cycle arrest at G2 phase was detected after IENS treatment with 5 μM of NC-GlaB at 24 and 48 h (∼25.3 and 30.2% of cells in G2, respectively), when compared with untreated cells (∼15.0 and 5.3%). Similar values were obtained for treatment with 5 μM of free GlaB (∼26.7 and 24.8%). In the case of the PANC1 cells (Figure 5, right panel and Supplementary Figure 4), a shift in cell cycle distribution from G1 to S phase was observed with GlaB treatments at 24 and 48 h (∼35.5 and 37.9% of cells in the S phase, respectively), compared with untreated cells (∼25.2 and 24.6%). A significant shift in the cell cycle profile was not detected in PANC1 cells incubated with NC-GlaB.

### Effect of GlaB treatment on Gli1 protein expression, analyzed by western blot

Since not enough protein could be obtained from IENS or G7 cells treated with GlaB, due to high sensitivity to the drug, PANC1 and SKOV3 were selected to further clarify the influence of GlaB incubation on Gli1 protein levels. In this regard, decreased Gli1 expression was detected in the cells after incubation with 5 μM GlaB for 24 h (∼73.3% relative expression for SKOV3, 88.1% for PANC1) or 48 h (∼33.6% for SKOV3, 44.6% for PANC1), compared with control untreated cells (100%) (Supplementary Figure 6). These results suggest that GlaB treatment suppresses the expression of Gli1.

### Effect of GlaB treatment in differentiated CSCs

**Figure 6.**
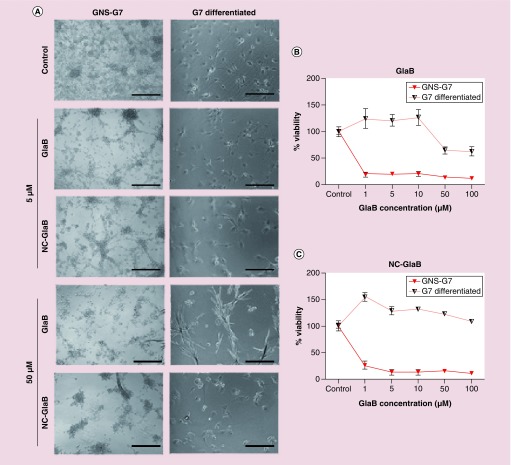
***In vitro* cytotoxicity of glabrescione B and glabrescione-B-loaded oil-cored polymeric nanocapsules in normal or differentiated G7 cells.** Cells were incubated with GlaB or NC-GlaB for 96 h at increasing drug concentrations (1–100 μM). **(A)** Microscopic observation of cells after treatment with GlaB or NC-GlaB for 96 h at 5 and 50 μM of GlaB (scale bar corresponds to 150 μm). Cell viability after 96 h of treatment with **(B)** free GlaB or **(C)** NC-GlaB was determined by MTT assay. Results, expressed as percentage to control untreated cells, correspond to mean ±SD of five different experiments. GlaB: Glabrescione B; NC: Nanocapsule; NC-GlaB: GlaB-loaded oil-cored polymeric nanocapsule; SD: Standard deviation.

In order to confirm that the activity of GlaB is Hh dependent (and thus connected to stemness), the differentiation of G7 cells into astrocytes was promoted by incubating these cells with BMP4, as shown previously by Carén *et al*. [35]. It is expected that differentiated G7 cells are less dependent on Hh pathway and therefore less sensitive to GlaB. Indeed, as shown in Figure 6A & B, and opposed to what was observed with pristine G7 cells, no significant toxicity was seen in differentiated G7 cells incubated with free GlaB for the tested concentrations except the highest (50 μM). This unexpected toxicity could be due to crystal formation at such high drug concentration. When incubated with NC-GlaB at all the tested concentrations, no significant cytotoxicity was observed in differentiated G7 cells (Figure 6A & C; Supplementary Figure 7).

### Radiolabeling of NC-PEG-GlaB & *in vitro* stability

**Figure 7.**
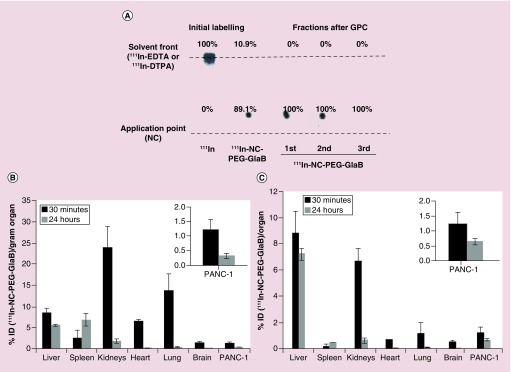
**Radiolabeling of NC-PEG with ^111^In, stability and *in vivo* biodistribution.** **(A)** TLC of the radiolabeled NCs immediately after radiolabeling and after desalting using PD10 desalting column, after concentrating the sample and just prior injection. No free ^111^In was detected in all the samples. A single dose of radiolabeled NCs was administered to SCID/Beige mice, via tail vein. **(B)** Percentage injected dose per gram of organ (% ID/g of organ) and **(C)** percentage injected dose per organ (% ID/organ), at 0.5 and 24 h after injection of 370 μg GlaB/mouse. Insets show the uptake in tumors. Data were expressed as means ± standard deviation (n = 2). GlaB: Glabrescione B; ID: Injected dose; NC: Nanocapsule.

In order to perform *in vivo* biodistribution studies, radiolabeled NCs were prepared using the previously synthesized PLGA–NH–PEG–NH–DTPA at 10% (w/w) of total polymer content. The presence of DTPA in the shell of the NCs is necessary for complexing ^111^In, a useful short-lived γ emitter used in biodistribution studies. The radiolabeling efficiency, calculated as the percentage of bound ^111^In of total added ^111^In, was tested using instant thin layer chromatography (TLC) technique after chelation of ^111^In. Only [^111^In]–EDTA or [^111^In]–DTPA chelates migrated to the solvent front while the radiolabeled polymer remained at the application point. Approximately 90% of radiolabeling efficiency was achieved for NC–DTPA (Figure 7A). Buffer exchange from ammonium acetate to PBS was also carried out using gel filtration chromatography. By passing the NC suspension through PD10 size exclusion columns, unbound ^111^In was retained in the column and the eluted NC suspension (in PBS) was ready for injection to mice. Radiolabeled NCs were found to be stable in solution, with approximately 92 and 72% stability obtained after NC incubation in PBS or 50% FBS (respectively) at 37°C for 24 h.

### Biodistribution of radiolabeled NC–PEG–GlaB in mice after single intravenous injection

The biodistribution of radiolabeled NC–PEG–DTPA–GlaB was investigated in pancreatic-tumor-bearing SCID/Beige mice. After receiving a single tail vein injection of NCs (∼370 μg GlaB per mouse), the animals were sacrificed at 30 min or 24 h postinjection and the % ID/g of tissue and % ID/organ were calculated. As shown in Figure 7B & C, NCs showed accumulation in major first-pass organs such as liver and spleen at 24 h after injection. Tumor uptake was 1.2 and 0.3% ID/g at 30 min and 24 h, respectively. Mice were not perfused before sacrifice. It is therefore likely that the higher % ID/g tumor obtained at 30 min than 24 h is due to high concentration of the drug in the blood.

### Histological examination of major organs following multiple injections of radiolabeled NC–PEG–GlaB in mice

**Figure 8.**
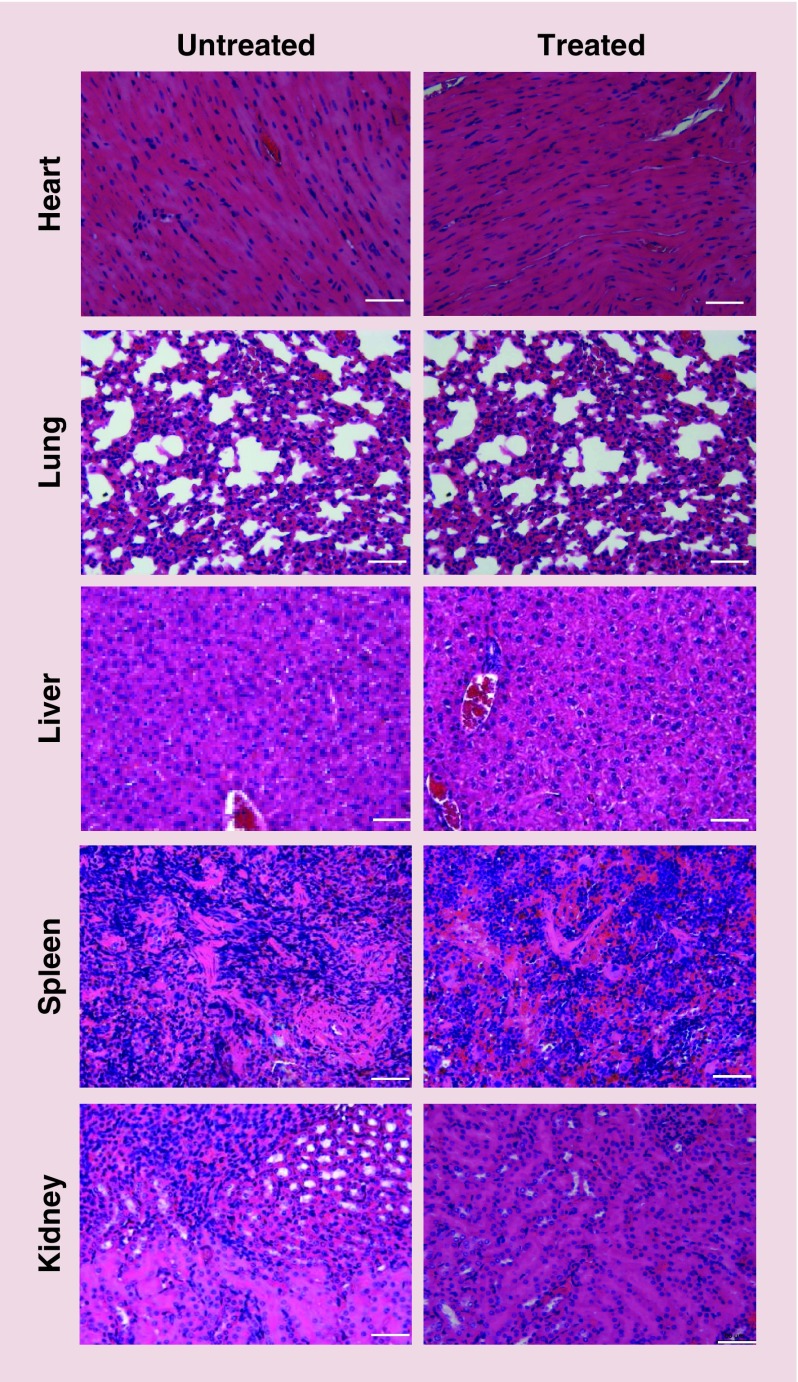
**Organ histology for NSG (NOD/SCID gamma) mice treated with multiple nanocapsule injections.** NSG mice were injected intravenously with 250 μg of NC-formulated GlaB once a week for 3 weeks. After the mice were sacrificed, the organs were immediately fixed in 10% neutral buffer formalin as 5 mm^2^ pieces. These pieces were then paraffin-embedded, sectioned for hematoxylin and eosin stains and imaged with light microscopy. Scale corresponds to 50 μm. GlaB: Glabrescione B; NC: Nanocapsule.

To evaluate the systemic toxicity of radiolabeled NC–PEG–DTPA–GlaB, pancreatic-tumor-bearing SCID/Beige mice were injected with the NCs (∼250 μg GlaB/mouse), via tail vein, once a week for 3 consecutive weeks. Histological analysis, carried out on hematoxylin and eosin stained tissue sections, did not show significant changes in lung, heart, kidney, liver and spleen compared with untreated (naive) tissues (Figure 8). This initially suggests that the NC–PEG–GlaB formulation can be administered via systemic injection. Extensive biocompatibility studies of this nanoformulation need to be assessed in the future.

## Discussion

CSCs are emerging as key therapeutic targets in oncology, as their efficient elimination may offer long-lasting tumor remission or even potentially disease-free treatment outcomes for patients. In this regard, a substantial amount of evidence suggests that CSCs rely on ligand–receptor-mediated signaling pathways, namely Hh, Wnt and Notch, for strict control of cellular functions that impart cell fate, namely proliferation, self-renewal and differentiation [44,45]. Aberrant Hh signaling is found in the majority of the human cancers, including brain tumors [18], and is usually caused not only by mutations in Hh pathway components (e.g., receptors) but also by high expression of Hh ligands [13], Hh signaling, which plays an important role in stem cell renewal, is epigenetically regulated in CSCs mainly Gli transcription factors [46]. Targeting Hh signaling components or epigenetic regulators in CSC-driven tumors could therefore be of therapeutic interest.

To date, an inhibitor of Hh pathway receptor (Smo) – GDC-0449 – has been approved by US FDA and is currently used in clinics, whereas only a few Gli inhibitors have been identified [47,48]. Therefore, novel drugs targeting Gli family members or downstream effectors would be beneficial for a wide spectrum of patients whose tumors have high Gli protein levels and/or activity. Furthermore, downstream activation of Gli function has been reported [49], with the development of resistance to Smo antagonists being observed frequently during therapy. Recent *in silico* and *in vitro* screens of a library of natural compounds identified a lead candidate for Gli inhibition – GlaB [22]. GlaB, bearing the 5,7-dimethoxyisoflavone nucleus, was isolated and characterized from the seeds of *D. glabrescens*, a Brazilian plant, in 1977 by Delle Monache *et al*. [21]. Since developed extraction methods allow us to get very limited amounts of pure isoflavone, here we provided the first total synthesis of GlaB which foresees a six-step route with an overall yield of 7%.

The *in vitro* and *in vivo* GlaB activities were also tested in our previous study, with results showing impaired Hh oncogenic activity via inhibition of Gli1/DNA interaction [22]. This provided proof of concept for the therapeutic relevance of an Hh-targeting approach focused on a downstream Gli effector rather than on upstream oncogenic deregulated signals. However, the native GlaB molecule is insoluble in water (solubility is 0.02 μg/ml), which will likely translate into poor bioavailability *in vivo*. In a previous study from our group, the efficacy of GlaB against MB-bearing and BCC-bearing mice was assessed [22]. A solution of GlaB, prepared in 2-hydroxypropyl-β-cyclodextrin:ethanol (3:1), was injected subcutaneously, compromising drug bioavailability. In this study, the use of ethanol was replaced with the long-circulating NC-GlaB, allowing intravenously injection of relatively high GlaB dose.

We have previously developed PEG-conjugated polymeric oil-cored NCs to enhance systemic delivery of therapeutic agents and active natural compounds as quercetin and curcumin [27,28]. Increased tumor uptake of folic-acid-conjugated NCs, containing quercetin, was observed after intravenous administration of in mice via passive and active targeting, while passively targeted curcumin-loaded PEG–PLGA NCs enhanced curcumin release into the tumor site after systemic administration, when compared with the free drug [28]. According to these studies, in an attempt to formulate NCs characterized by smallest size, narrowest PDI, higher colloidal stability and drug loading, different oil-cored types were used [28]. Among all, castor oil was found to be the one capable of obtaining the desired NCs [28]; thus, it has been selected as the most promising system to enhance GlaB bioavailability. In particular, GlaB was successfully loaded into NCs composed of castor-oil-cored offering approximately 70-fold increase in GlaB aqueous solubility (solubility in NC: ∼700 μg GlaB/ml of NC). Regarding the choice of the polymer, different PLGA polymer derivatives were used depending on the experiment that needed to be performed. Both PEG–PLGA and DTPA–PEG–PLGA were obtained through organic synthesis [28] and, in accordance with the previous studies [27,28], the conjugation of PLGA with PEG leads to a slight increase (less negative) in surface charge of NC–PEG and NC–PEG–GlaB, probably due to neutralization of the negative carboxylate groups of PLGA (Table 1 and Supplementary Table 1). Importantly, empty NCs and NC-GlaB showed similar hydrodynamic size and stability over time, which suggests that the encapsulation of GlaB does not affect NC size and long-term storage. Comparable biological evaluation results were obtained for both NC and NC-PEG (Supplementary Figure 4) when tested in IENS at the three time points. The good *in vitro* serum stability data (<20% release over 24 h) suggest that there will be minimal drug release from the NC in blood circulation and that most of the drug will be released at the target site, in other words, the tumor.

A series of *in vitro* functional assays were then performed to evaluate and compare the anticancer properties of NC-GlaB and free GlaB. Cell lines for these studies were chosen, after literature consultation, on the basis of Hh status (dysregulated Hh pathway) and presence of stem-like cells in the cell population. *In vitro* results obtained from cell viability, cell cycle analysis and western blotting confirmed that CSC cell lines (G7, IENS) were more sensitive to GlaB than non-stem cancer cells (GL261, PANC1 and SKOV3), an effect that was mediated by Hh regulator Gli, which is more pronounced in CSCs. This was further confirmed by the fact that differentiated G7 cells were drug insensitive. Future studies will be performed to prove unequivocally that Hh signaling is dysregulated in the CSCs used in this study (G7, IENS).

The fact that GlaB was effective in killing CSCs at very low concentrations (∼70% cell killing with 1 μM for 48 h) is very encouraging and suggests a promising *in vivo* therapeutic outcome if such concentrations can be achieved *in vivo* following systemic injection. Moreover, these results are consistent with previous *in vitro* studies, in which GlaB (5 μM) was shown to inhibit proliferation of *ex vivo* cultured mouse granule cell progenitors, MB stem cells isolated from Ptch1^±^ mice tumors and ASZ001 BCC cells, with a strength similar to GANT61 (10 μM) [22]. Indeed, GANT61 has become the reference compound for CSC-targeted therapies as it proved to inhibit the Hh pathway at Gli level in several cancer cells, CSCs and tumor animal models, such as pancreatic [17], prostate [14], lung [12], colon and hepatocellular cancer [50,51], as well as in tumors for which the role of Gli was not elucidated before, including ovarian cancer [52], esophageal adenocarcinoma and melanoma [8,53]. However, GANT61 inhibits Gli-mediated transcription by an unclear mechanism – most likely by inducing post-translational modifications of Gli – with a reported IC_50_ of 5 μM (in HEK293 cell line). In our study, GlaB and NC-GlaB were shown to be more effective than GANT61 in inducing tumor cell death, with IC_50_ values in PANC1 of 1.62 and 1.49 μM, respectively.

Following up the positive *in vitro* studies, it was necessary to overcome the poor water solubility and bioavailability of GlaB so that it can be validated for clinical application. Our previous studies assessed organ distribution of PEG–NCs in a leaky solid tumor model – CT26 colon cancer – and approximately 0.8% ID/g of tissue in tumors was achieved 24 h postinjection [28]. Furthermore, curcumin, used as an anticancer drug in that study, has shown to be delivered to the tumor at therapeutic concentrations leading to significant tumor growth delay [28]. In preparation for preclinical testing of therapeutic activity, we first assessed organ biodistribution in PANC1-tumor-bearing mice. This tumor model is known to be highly resistant and not sufficiently leaky [17]. Such data will give us some guidance on the next stages required to prepare glioma-targeting NC formulation to deliver GlaB to brain cancer. Our results have shown that values of 0.5–1.25% ID/g tissues were achieved in tumors 24 h postinjection. Taking into account the fact that the dose administered was approximately 370 μg GlaB per mouse, one would expect that drug concentrations in the tumor to be approximately 1.9–4.4 μg/g tumor (4–10 nM). These concentrations are below the therapeutic concentrations used in PANC1, and it is therefore suggested that higher or multiple doses of the formulation is required.

The results obtained from this work gave us some guidance on the next stages, to use NCs in a leaky tumor. We are also currently working on reducing the size of our NCs to less than 150 nm as smaller sizes are expected to have better extravasation than 200 nm NCs, before testing them in other tumor models for therapy. Moreover, the therapeutic efficacy of these NCs will be tested against CSCs isolated from other types of tumor, such as pancreas, or Hh-driven tumors such as MB.

## Conclusion

The efficient encapsulation of GlaB into oil-cored NCs (NC-GlaB) greatly improved the bioavailability and potential clinical application of this drug. More importantly, NC-GlaB retained the same antitumoral activity of the free drug, both revealing remarkable cell killing activity against Hh-dependent CSC lines and, to a lesser extent, non-stem cancer cells. Substantial evidence is provided for a nanoparticle-based drug with good circulation profile that could be useful as part of a multimodal treatment for patients affected by Hh-dependent CSC-driven tumors.

**Executive summary**

**Background**

- Use of PEG-conjugated polymeric oil-cored nanocapsules (NCs) to enhance systemic delivery of glabrescione B (GlaB).

**Synthesis of GlaB**

- The total synthesis of GlaB in six steps with an overall yield of 7% is reported for the first time.

**Physico-chemical characterization of NCs**

- GlaB was successfully loaded into NCs composed of castor-oil-cored NCs offering approximately 70-fold increase in GlaB aqueous solubility.
- Hydrodynamic diameter, polydispersity index, ζ potential and stability were determined.
- *In vitro* serum stability data suggest that there will be minimal drug release from the NC in blood circulation and that most of the drug will be released at the target site, in other words, the tumor.

***In vitro* functional assays**

- Cell viability, cell cycle analysis and western blotting confirmed that cancer stem cell lines (G7, IENS) were more sensitive to GlaB than non-stem cancer cells (GL261, PANC1 and SKOV3).
- Differentiated G7 cells were drug insensitive as they could be less dependent on Hh pathway.

***In vivo* biodistribution**

- The biodistribution of radiolabeled NC-GlaB was investigated in pancreatic-tumor-bearing SCID/Beige mice and 0.5–1.25% ID/g tissues were achieved in tumors at 24 h postinjection.
