# Supplementary material for: Polymeric glabrescione B nanocapsules for passive targeting of Hedgehog-dependent tumor therapy in vitro
Source: Nanomedicine (Lond). 2017 Mar 21;12(7):711–28. doi: 10.2217/nnm-2016-0388 (PMC5986025; doi:10.2217/nnm-2016-0388)
Supplement: Supplementary file 1 [file nnm-12-711-s1.docx]

**Supplementary Information**

**Polymeric glabrescione B nanocapsules for passive targeting of Hedgehog-dependent tumor therapy *in vitro***

**Keywords:** Cancer stem cells (CSCs), Gli inhibitor, Oil-cored polymeric nanocapsules.

**Supplementary Materials**

*Equipment used during GlaB synthesis*. Chromatography was carried on silica gel (70−230 mesh). All reactions were monitored by thin-layer chromatography (TLC), and silica gel plates with fluorescence F254 were used. Melting points were taken in open capillaries on a BüCHI Melting Point B-545 apparatus and are presented uncorrected. Infrared spectra were recorded in KBr pellets or neat on NaCl on a FT-IR spectrometer. ^1^H NMR and ^13^C NMR spectra were recorded using a Bruker 400 Ultra Shield^TM^ spectrometer (operating at 400 MHz for ^1^H and 100 MHz for ^13^C) using tetramethylsilane (TMS) as internal standard. Chemical shifts are reported in parts per million. Multiplicities are reported as follows: singlet (s), doublet (d), triplet (t), multiplet (m), broad singlet (brs) and broad quadruplet (brq). Signals for -N*C*H_3_ carbon are not present in the ^13^C NMR data. Mass spectrometry was performed using a Thermo Finnigan LXQ linear ion trap mass spectrometer, equipped with an electrospray ionization (ESI) ion source bearing a steel needle. High resolution mass spectra (HRMS) were recorded on Bruker BioApex Fourier transform ion cyclotron (FT-ICR).

**Supplementary Results**

**Table S1**. **Shelf-life stability of oil-cored prepared using PLGA-PEG as polymer.**

| Sample | Day | Hydrodynamic diameter [nm] ^a,e^ | PDI ^a,e^ | Zeta-potential [mV] ^b,e^ | Encapsulation Efficiency  [EE%] ^c,e^ | Loading Efficiency [LE%] ^d,e^ |
| --- | --- | --- | --- | --- | --- | --- |
| NC | 1 | 182.2 ± 1.0 | 0.23 ± 0,01 | -34.0 ± 2.0 | - | - |
|  | 7 | 155.5 ± 0.7 | 0.11 ± 0.01 | -46.6 ± 3.2 |  |  |
|  | 14 | 167.3 ± 2.4 | 0.18 ± 0.01 | -34.0 ± 1.4 |  |  |
|  | 21 | 152.5 ± 0.9 | 0.13 ± 0.02 | -46.2 ± 1.2 |  |  |
|  | 28 | 153.0 ± 4.2 | 0.11 ± 0.01 | -44.8 ± 2.0 |  |  |
| NC-GlaB | 1 | 164.9 ± 0.5 | 0.19 ± 0.01 | -42.0 ± 2.3 | 85% ± 6.1 | 27.5 ± 1.9 |
|  | 7 | 164.5 ± 1.2 | 0.18 ± 0.01 | -40.2 ± 2.4 |  |  |
|  | 14 | 162.3 ± 2.2 | 0.18 ± 0.01 | -40.5 ± 1.0 |  |  |
|  | 21 | 164.3 ± 2.2 | 0.19 ± 0.01 | -41.9 ± 0.1 |  |  |
|  | 28 | 151.2 ± 0.6 | 0.15 ± 0.01 | -42.7 ± 0.4 |  |  |

^a)^ Measured with dynamic light scattering.

^b)^ Analysed with electrophoretic light scattering using 10 mM NaCl.

^c)^ Calculated as percentage of initial drug added, which was determined by spectrophotometry.

^d)^ Calculated as mass of incorporated drug divided by the weight of polymer, which was determined by spectrophotometry.

^e)^ Expressed as mean ± SD (n=3).


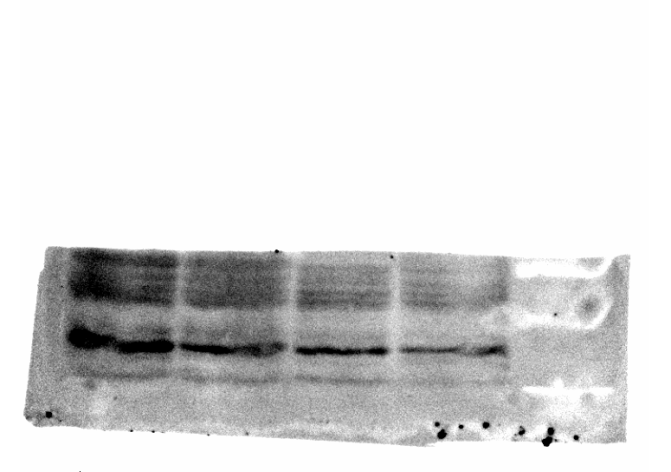

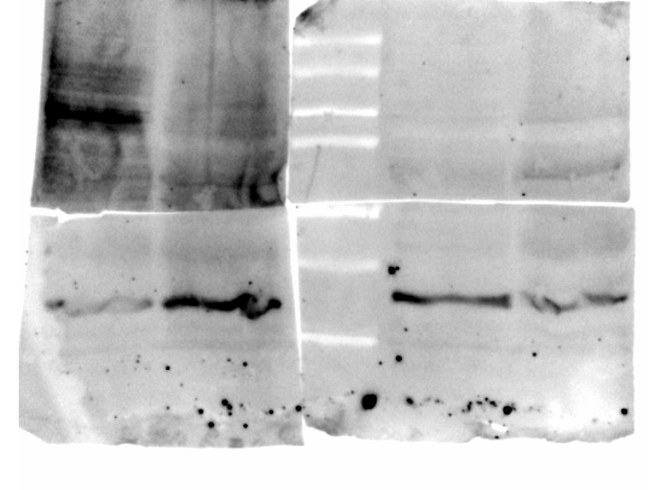

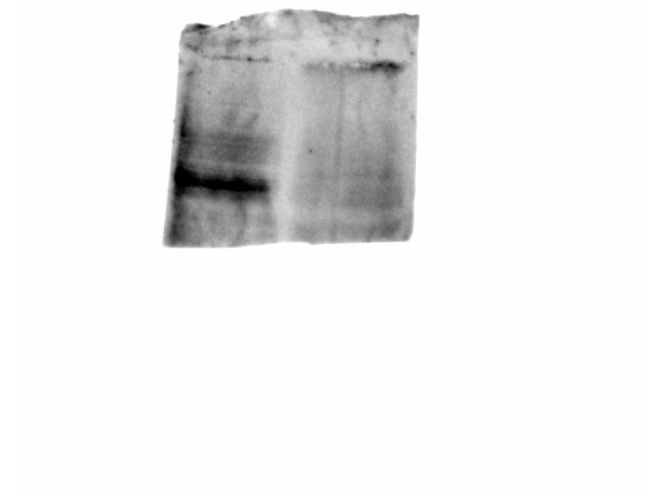

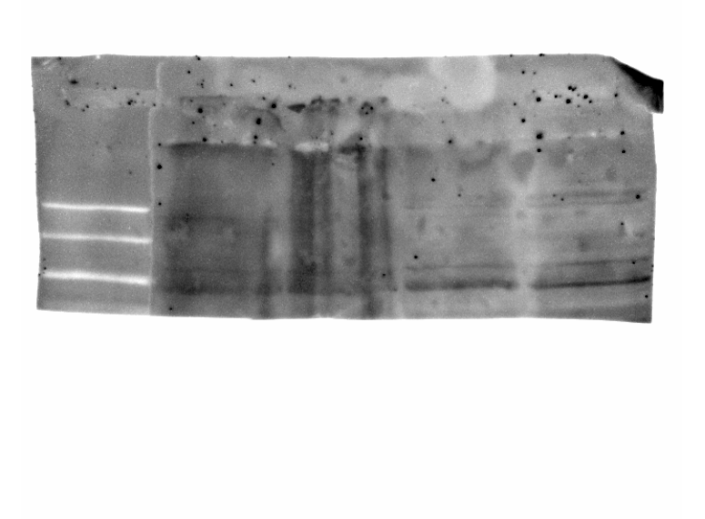

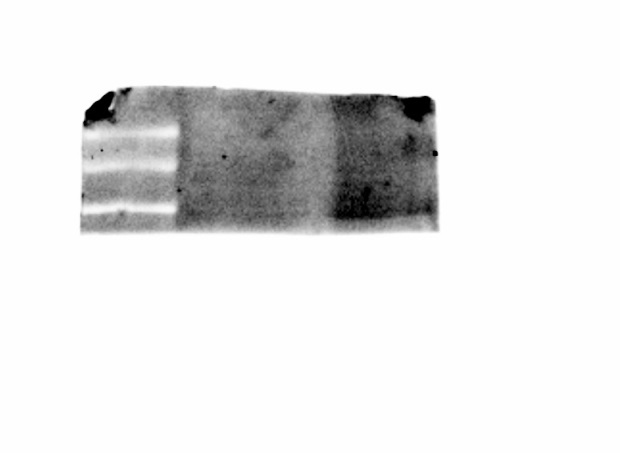

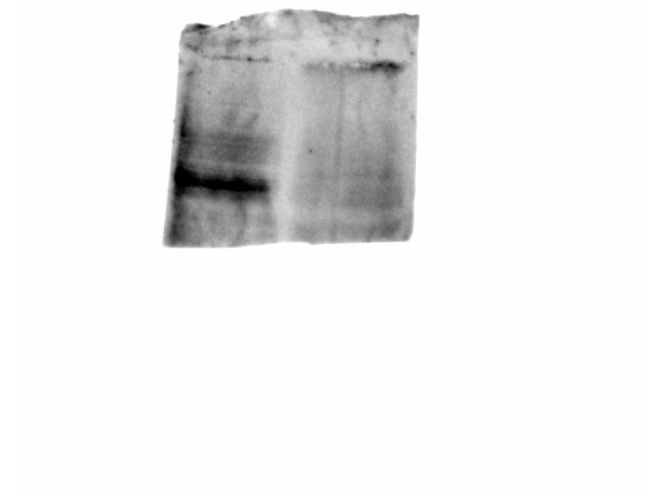

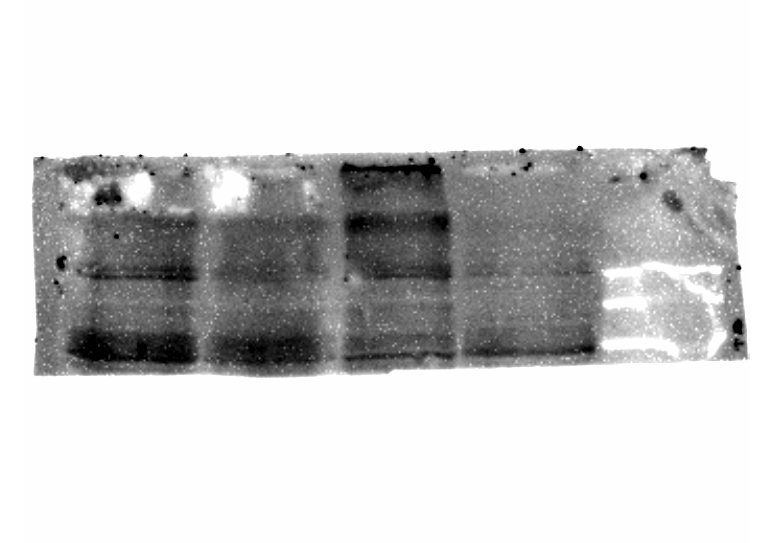

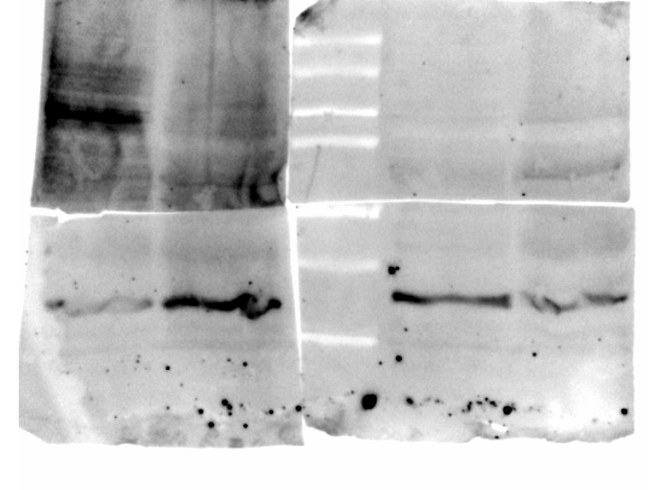

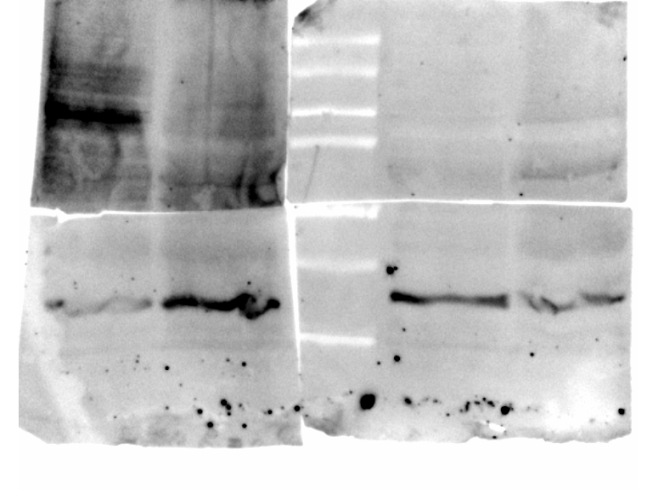


**Gli1**

**GAPDH**

**IENS**

**G7**

**PANC1**

**SKOV3**

**GL261**

F**ig. S1. Western blot analysis of Gli1 protein expression in different cell lines.** Gli1 protein levels in IENS, G7, PANC1, SKOV3 and GL261 cells. Gli1 protein levels were normalized to GAPDH expression and the results are expressed as relative Gli expression (%) compared to that of IENS cells.

**Fig. S2. *In vitro* cytotoxicity of GlaB and NC-GlaB.** Cells were incubated with GlaB or NC-GlaB at increasing drug concentrations (1 - 100 μM). **(A, B)** Cell viability expressed as a percentage of control untreated cells, determined by MTT assay, at **(A)** 48 and **(B)** 72 h treatment with free GlaB (left) or NC-GlaB (right). Results are expressed as mean ±SD (n= 5).

**Fig. S3. *In vitro* cytotoxicity of empty NCs.** Cells were incubated with empty NCs for (**A**) 48, (**B**) 72 or (**C**) 96 h, at increasing NC concentrations (expressed as µg/mL of polymer) equivalent to those used at 1 - 100 μM GlaB. Results are expressed as mean ±SD (n= 5).

**Fig. S4. *In vitro* cytotoxicity of NC-PEG-GlaB or empty NC-PEG in the GNS-IENS cell line.** **(A, B)** Cells were incubated with NC-PEG-GlaB or NC-PEG for 48, 72 or 96 h at increasing concentrations of **(A)** GlaB (1 – 100 μM) or **(B)** empty NCs (15,6, 78 and 156 μg/ml of the polymer, equivalent to 5, 50 and 100 μM GlaB concentrations). Cell viability was determined by MTT assay and expressed as percentage to control untreated cells. Results are expressed as mean ± SD (n= 5).

**Fig. S5. Cell cycle analysis in IENS and PANC 1 cells.** **(A)** Representative histograms showing the cell cycle distribution and **(B)** relative changes in the percentage of cells in each cell cycle phases, following 24 h treatments with 5 μM GlaB. Results are expressed as mean ± SD (n= 3).


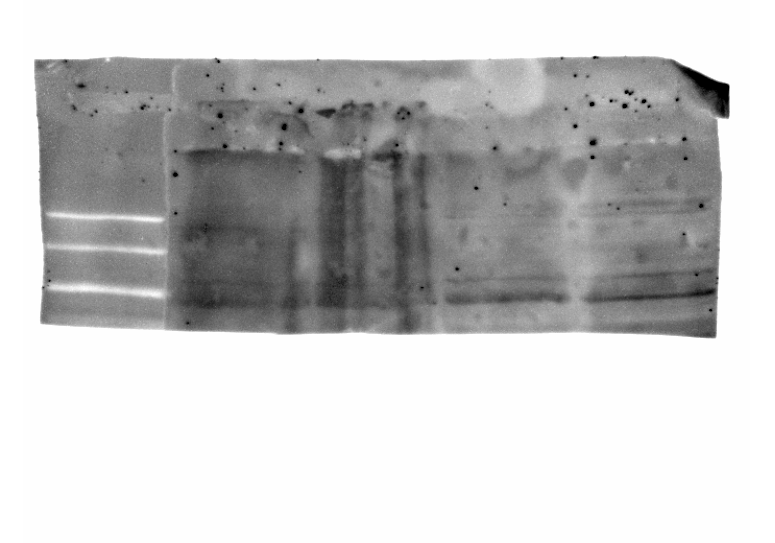

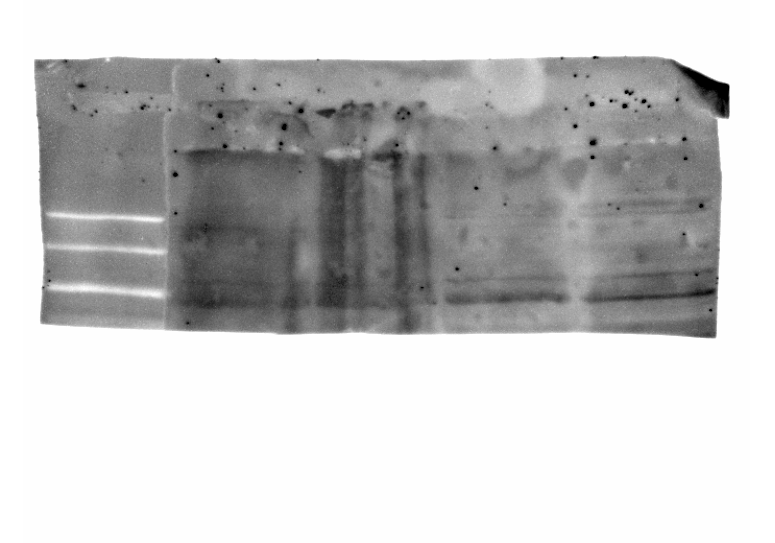

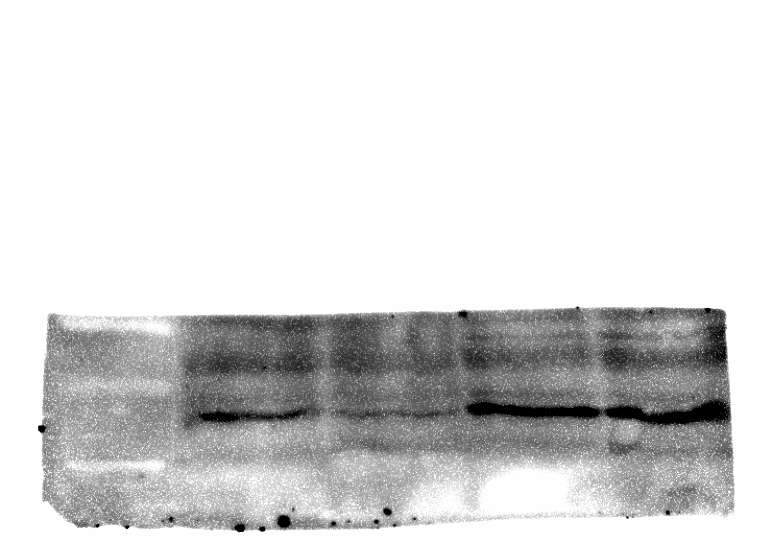

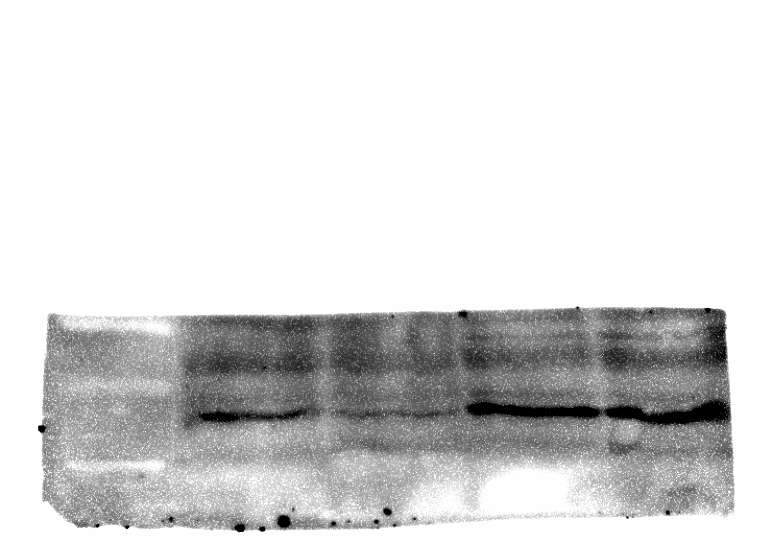

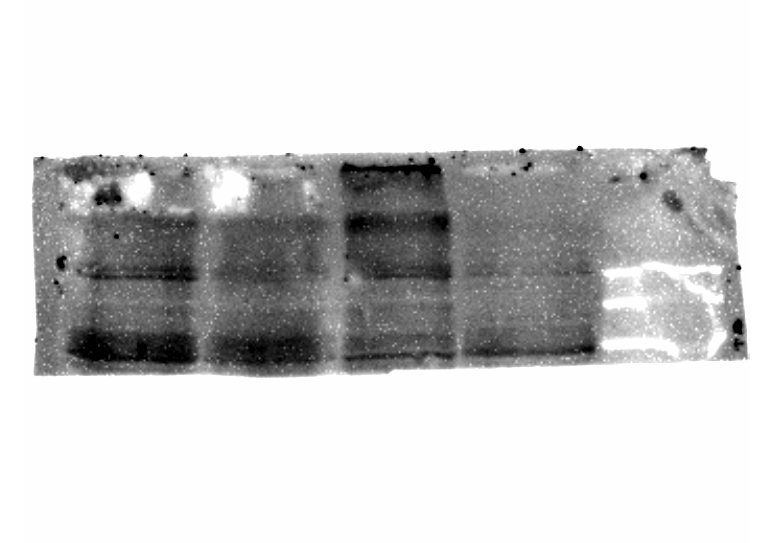

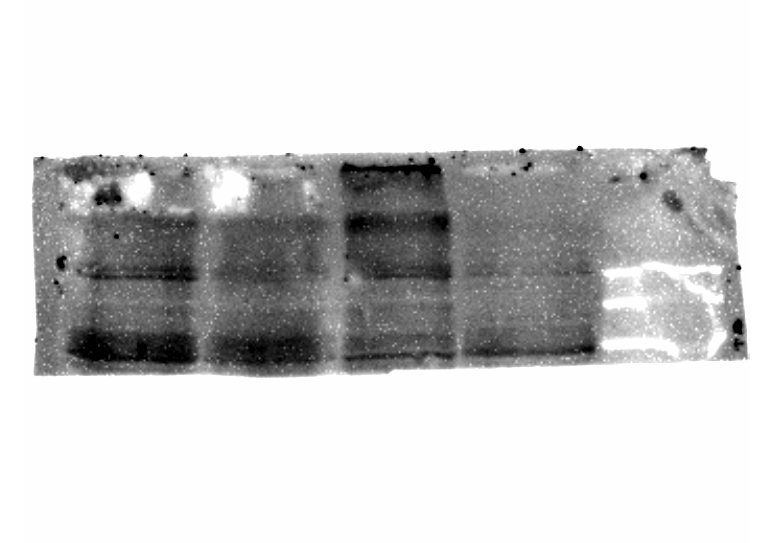

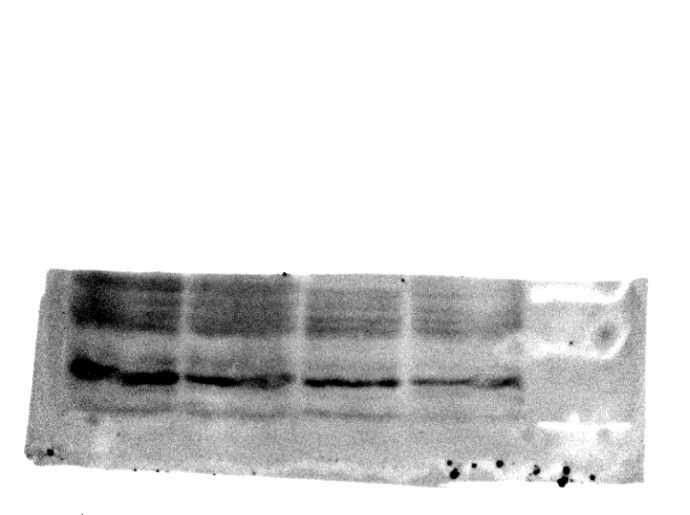

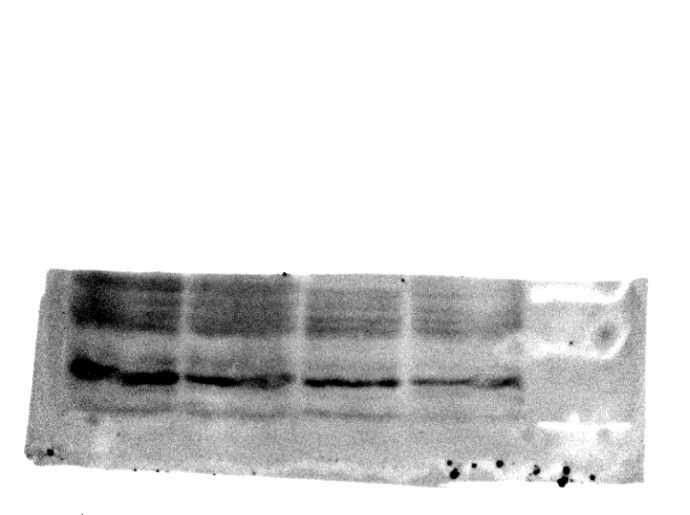

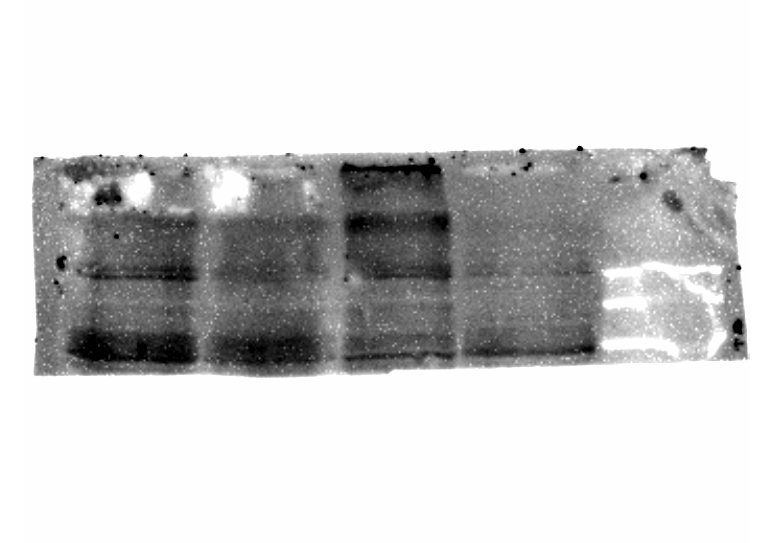


**Gli1**

**GAPDH**

**Ctr**

**24h**

**48h**

**Ctr**

**24h**

**48h**

**PANC1**

**SKOV3**

**Fig. S6. Western blot analysis of Gli1 protein expression in PANC1 and SKOV3 after GlaB treatment.** Gli1 protein levels in PANC1 and SKOV3 cells after treatment with 5 µM NC-GlaB for 24 or 48 h. Gli1 protein levels were normalized to GAPDH expression and the results are expressed as % Gli expression compared to untreated cells.

**Fig. S7. Cytotoxicity of GlaB and NC-GlaB in normal or differentiated G7 cells *in vitro*.** Cells were incubated with GlaB (left) or NC-GlaB (right) for **(A)** 48 h or **(B)** 72 h at increasing drug concentrations (1 - 100 μM). Cell viability expressed as a percentage of control untreated cells, as determined by MTT assay. Results are expressed as mean ±SD (n= 5).

**Fig. S8. GlaB quantification with UV spectroscopy or HPLC-UV-Vis.**  Linear regression plots of different GlaB concentrations **(A)** from 1 to 20 µg/mL in ethanol at 258 nm by with spectroscopy or **(B)** from 1 to 8 µg/mL in acetonitrile with HPLC-UV-Vis. GlaB peak is observed at retention time of 28.1 min. Results are expressed as means ± SD (*n*=3).
